# Molecular detection and identification of *Culex* flavivirus in mosquito species from Jeju, Republic of Korea

**DOI:** 10.1186/s12985-021-01618-9

**Published:** 2021-07-19

**Authors:** Shilpa Chatterjee, Choon-Mee Kim, Na Ra Yun, Dong-Min Kim, Hyeon Je Song, Kyeoung A Chung

**Affiliations:** 1 Department of Internal Medicine, College of Medicine, Chosun University, 588 Seosuk-dong, Dong-gu, Gwangju, 61453 Republic of Korea; 2 Department of Premedical Science, College of Medicine, Chosun University, Gwangju, Republic of Korea; 3 Department of Clinical Laboratory Science, Gwangju Health University, Gwangju, Republic of Korea

**Keywords:** Flavivirus, Insect-specific flavivirus, *Culex pipiens*, Nonstructural protein 5, Reverse transcriptase nested polymerase chain reaction, Jeju province

## Abstract

**Background:**

Mosquito-borne flaviviruses are prime pathogens and have been a major hazard to humans and animals. They comprise several arthropod-borne viruses, including dengue virus, yellow fever virus, Japanese encephalitis virus, and West Nile virus. *Culex* flavivirus (CxFV) is a member of the insect-specific flavivirus (ISF) group belonging to the genus *Flavivirus*, which is widely distributed in a variety of mosquito populations.

**Methods:**

Viral nucleic acid was extracted from adult mosquito pools and subjected to reverse transcriptase nested polymerase chain reaction (PCR) using target-specific primers for detecting CxFV nonstructural protein 5 (NS5). The PCR-positive samples were then sequenced, and a phylogenetic tree was constructed, including reference sequences obtained from GenBank.

**Results:**

21 pools, belonging to *Culex pipiens pallens* (*Cx. p. pallens)* were found to be positive for the CxFV RNA sequence, with a minimum infection rate of 14.5/1000 mosquitoes. The phylogenetic analysis of the NS5 protein sequences indicated that the detected sequences were closely related to strains identified in China, with 95–98% sequence similarities.

**Conclusion:**

Our findings highlight the presence of CxFV in *Cx. p. pallens* mosquito species in Jeju province, Republic of Korea. This is the first study reporting the prevalence of CxFV in *Culex Pipiens (Cx. pipiens)* host in the Jeju province, which can create possible interaction with other flaviviruses causing human and animal diseases. Although, mosquito-borne disease causing viruses were not identified properly, more detailed surveillance and investigation of both the host and viruses are essential to understand the prevalence, evolutionary relationship and genetic characteristic with other species.

## Background

Mosquito-borne flavivirus infection has been a major hazard to human health until the twenty-first century, particularly in tropical and subtropical regions [1]. The genus *Flavivirus* belongs to the family *Flaviviridae*, which comprises approximately 73 arthropod-borne viruses such as dengue virus (DENV), yellow fever virus (YFV), Japanese encephalitis virus (JEV), and West Nile virus (WNV), which infect rodents, pigs, humans, and other mammalian hosts. Flaviviruses are the most prevailing arthropod-borne viruses and among them many are identified as human pathogens. Flaviviruses shows similarity in genomic organizations but differ in transmissibility and their host ranges [2]. Most of them are dual-host flaviviruses that possess horizontal transmission between vertebrate hosts and arthropod vectors. However, not all flaviviruses are dual-host in nature; some are vertebrate-specific (No known vector virus) and others are insect-specific flavivirus (ISFs) that replicate only in insect cells and not in vertebrate cells [3].

Culex flavivirus (CxFV) is an ISF that was first identified in 2003–2004 in *Culex* species in Japan and Indonesia [4]. CxFV was later identified in field mosquito populations in many other countries which lead to the discovery of several distinct ISFs in the last few years [5, 6]. Though many flaviviruses are still endemic in tropical areas [7], the topographical alteration, rapid urbanization, and extensive deforestation have contributed to the increased prevalence of these pathogens in previously non-endemic areas [8, 9]. This, in turn, forms the basis for a wide variety of clinical indications, such as undifferentiated fever and encephalitis, which may potentially lead to death.

The objective of the study was to investigate the presence of flaviviruses in the mosquitoes of Jeju region using broad-spectrum primers targeting the NS5 gene. Further, we aimed to detect the presence of ISF CxFV in these mosquito species.

Here, we reported the abundance of vector mosquito *Cx. pipiens,* carrying the ISF CxFV in urban areas of Jeju province of the Republic of Korea (ROK) during 2018. This study could provide basis for further detailed investigation on the prevalence, evolutionary relationship and genetic characteristic with other species.

## Materials and methods

### Collection of mosquitoes

**Fig. 1:**
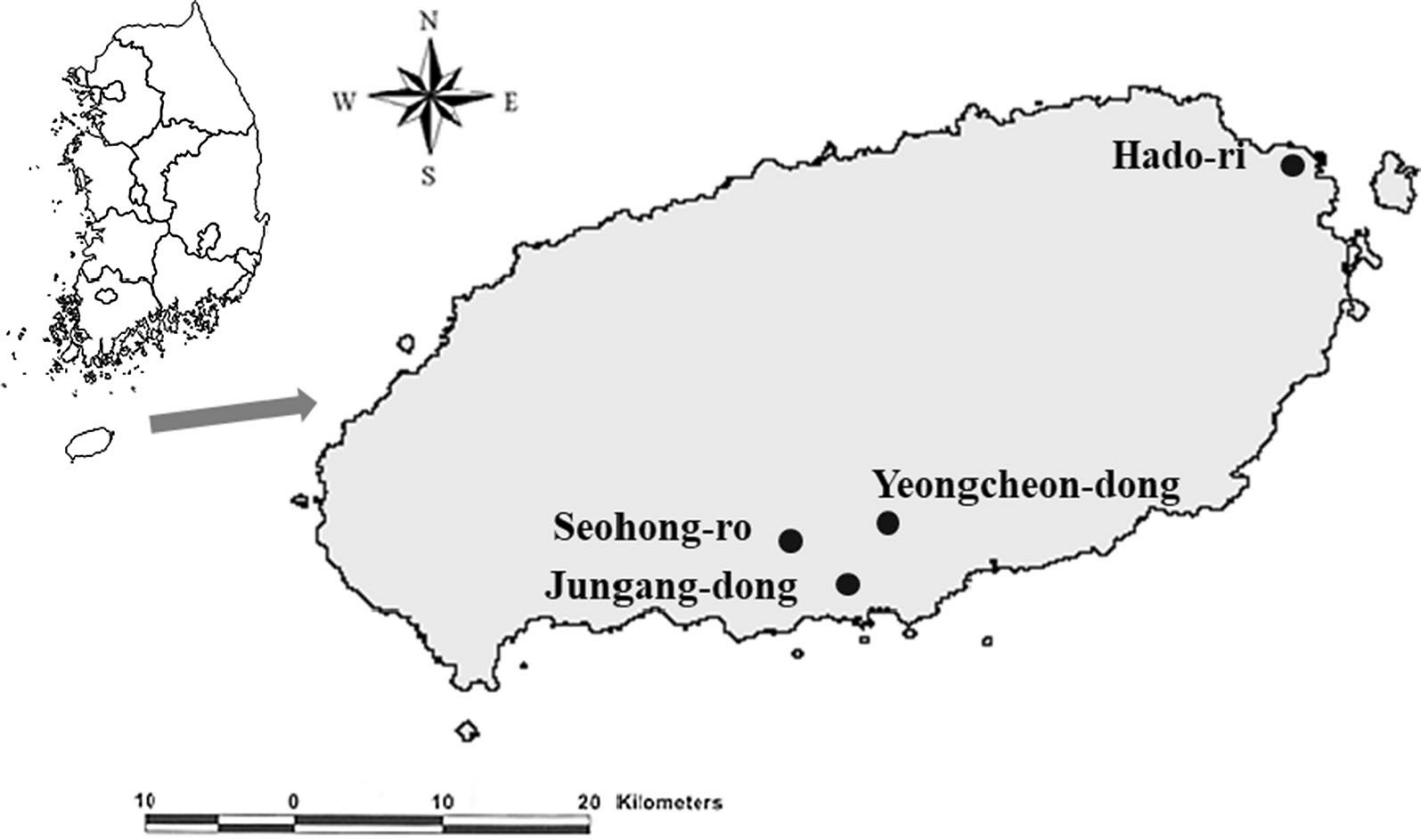
Geographical location of mosquitoes capturing sites: Hado-ri, Seohong-ro, Yeongcheon-dong, and Jungang-dong in Jeju province, Republic of Korea. Black dots mark the mosquito collection sites

Adult mosquitoes were collected monthly from March to November 2018 using BL (black light trap) and BG (Biogents' Sentinel 2 Mosquito Trap) traps at several locations in the Jeju region: Hado-ri (33.5150° N, 126.8818° E on Jeju Island), Seohong-ro (33.2671° N, 126.5486° E on Jeju Island), Yeongcheon-dong (33.2688° N, 126.5870° E on Jeju Island), and Jungang-dong (33.2507° N, 126.5651° E). The region was divided into cattle sheds, habitats of migratory birds, and downtown areas (Fig. 1).

Following field trapping, all collected mosquitoes were examined visually and microscopically to identify female mosquitoes, and kept at 6ºC until being transferred to the laboratory [10, 11]. The mosquitoes were divided into several pools according to their species, date, and place of collection. The identification and confirmation of the female mosquitoes were done morphologically with the help of specialized taxonomic keys [12, 13]

A total of 1877 mosquitoes were grouped into 207 mosquito pools (1–40 mosquitoes in each pool) belonging to 13 distinct species of the genera *Culex*, *Ochlerotatus*, *Anopheles*, *Mansonia*, *Armigeres*, and *Aedes*. The mosquito samples were stored at − 80 °C before being processed for further molecular detection.

### Nucleic acid extraction and polymerase chain reaction (PCR) amplification

A BioSpec Mini-BeadBeater 16 (Bio Spec Products Inc.; Bartlesville, OK, USA) was used to homogenize the sample pools with glass beads. Sterile phosphate-buffered saline (PBS, 800 µl) was added to each sterile micro beading tube (2 mL) containing a sample pool and homogenized. The homogenates were then centrifuged at 13,000 rpm for 1 min and 140 µL supernatant was used for viral RNA extraction using the QIAamp 96 Virus QIAcube HT kit (QiagenSciences; Germantown, MD, USA) in accordance with the manufacturer’s instructions.

Then, cDNA was synthesized using the extracted RNA as a template. The synthesized cDNA was then used in reverse transcription-based nested PCR (RT-nPCR) with primers detecting the NS5 partial gene specific to the flavivirus (212 bp) [14]. Two sets of primers were used: PanF-NS5-1373F and PanF-NS5-2481R for the initial amplification, and FL-F1, FL-R3, and FL-R4 to obtain a 212 bp product.

An AB thermal cycler (Applied Biosystems; Foster City, CA, USA) was used for PCR amplification. The reaction mixture comprised 20 μL solution including AccuPowerR PCR PreMix (Bioneer Corp.), 1 μL 10 pmol/μL primers (forward and reverse), 2 μL template cDNA (for the first PCR) or first PCR product (for the second PCR), and 16 μL sterile distilled water. A suitable positive control (DENV, Zika virus, YFV, and tick-borne encephalitis virus) and molecular grade water (as a negative control) were included in each run.

All primers used for the specific target gene and PCR cycling conditions along with the product sizes are given in Table 1.

**Table 1:** Polymerase chain reaction (PCR) conditions for the detection of flavivirus

Flavivirus Target gene	Primer name	Nucleotide sequence (5′-3′)	Product size (bp)	PCR protocol (˚C/s)				Reference
				Denaturation	Annealing	Extension	Cycles	
NS5	PanF-NS5-1373F PanF-NS5-2481R	AACATGATGGGVAARAGAGAG ATCCACACHCKGTTCCASAC	1108	94/60	52/60	72/60	35	In-house design
	FL-F1 FL-R3 FL-R4	GCCATATGGTACATGTGGCTGGGAGC GTKATTCTTGTGTCCCAWCCGGCTGTGTCATC GTGATGCGRGTGTCCCAGCCRGCKGTGTCATC	212	94/30	58/30	72/30	30	[14]

A consensus region of the NS5 gene was targeted by two sets of flavivirus-specific primers [14] for the detection of all flaviviruses. Here, the amplification products were expected to be 212 bp long. The amplicons were separated by electrophoresis using a 1.5% agarose gel and visualized by ethidium bromide staining. Furthermore, all PCR-positive products were subjected to nucleotide sequencing.

### Nucleotide sequencing, phylogenetic analysis, and calculation of the minimum infection rate (MIR)

To sequence the nucleotides of the amplified region for flaviviruses, PCR-positive samples were purified using a QIAquick Gel Extraction Kit (QIAGEN; Hilden, Germany). The amplicons from mosquito samples were sequenced by Cosmo Genetech (Daejeon, ROK). The Basic Local Alignment Search Tool (BLAST) was used to compare the obtained sequences to the GenBank deposited sequences. Default search parameters were used [15].

NS5 gene sequences were obtained from GenBank, and phylogenetic trees were constructed using Mega X [16] based on the alignments of positive gene sequences using the maximum likelihood (ML) method [17]. The tree is drawn to scale, with branch lengths measured in the number of substitutions per site. The percentage of replicate trees in which nodes were recovered under the bootstrap analysis (1,000 replicates) was calculated. Minimum infection rate (MIR) was calculated which was used as virus activity index in mosquito populations. The MIR was calculated using equation [18]:

$${\text{MIR}} = \left( {{\text{Number of positive pools}}/{\text{total number of mosquitoes tested}}} \right) \times 1000$$

## Results

A total of 1877 mosquitoes belonging to 13 different species, including four species of *Culex*, two species of *Anopheles* and *Aedes,* three species of *Ochlerotatus*, and single species of *Mansonia* and *Armigeres* were captured using BG and BL traps at four trapping sites on Jeju Island, ROK between March and November 2018 (Table 2).

**Table 2:** Number of mosquitoes and species collected during March to November 2018 in Jeju province, Republic of Korea

Month	No. of mosquitoes collected	No. of species collected
March	19	1
April	79	4
May	74	8
June	436	10
July	202	7
August	396	9
September	170	7
October	290	7
November	211	4

**Fig. 2:**
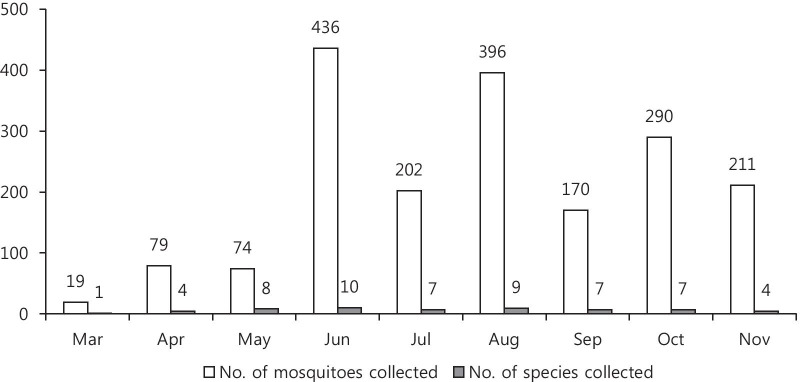
Month-wise distribution of mosquitoes and species collected during March to November 2018

Most mosquitoes were captured in June, August, and October (Fig. 2). The most predominant species detected were *Culex* species (77.2%). All trapped mosquitoes were female.

**Fig. 3:**
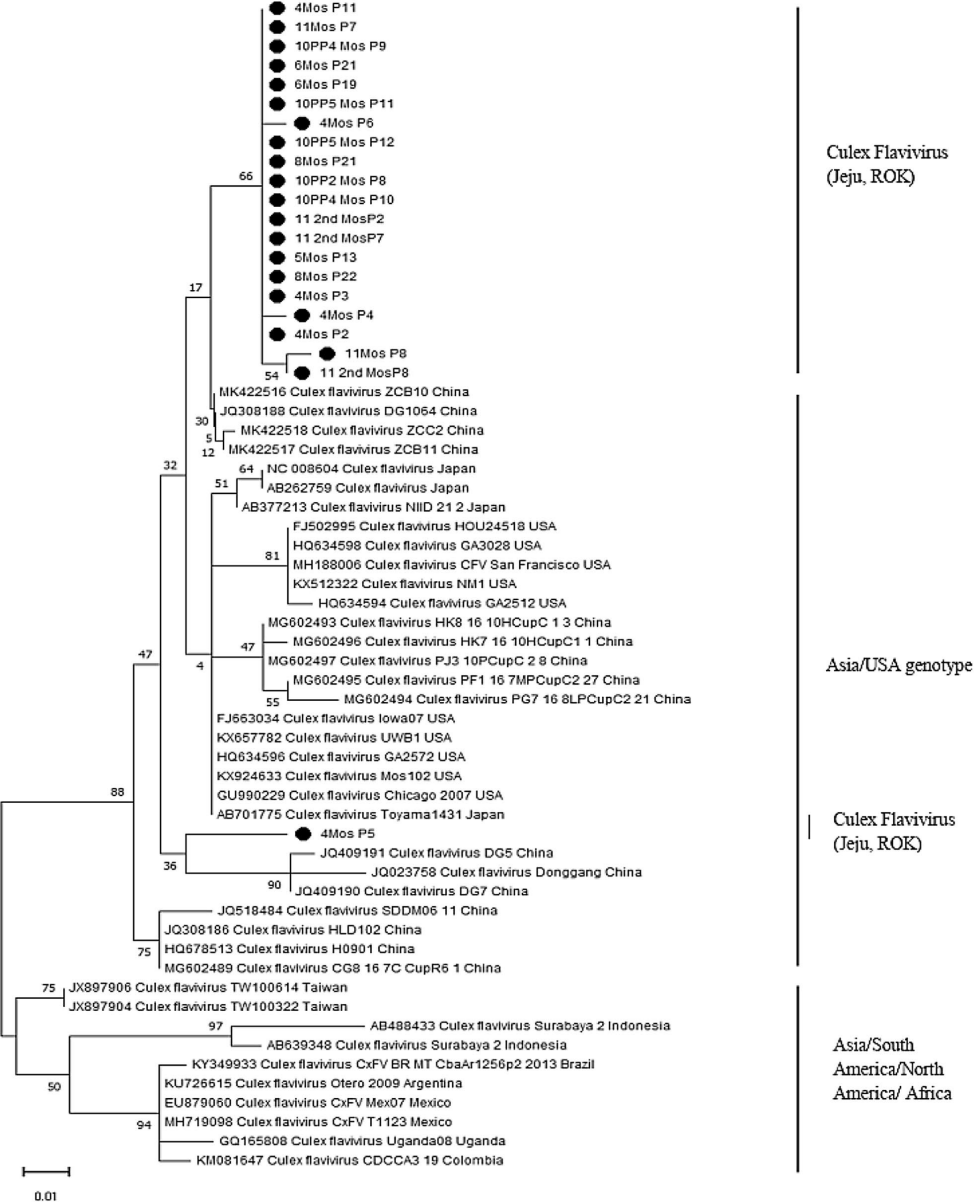
Phylogenetic tree was constructed based on the NS5 target gene (212 bp) of mosquito samples positive for Culex flavivirus (filled circle) and flavivirus sequences from GenBank.. See Table 4 for the GenBank accession numbers of Culex flavivirus sequences (Jeju, ROK).The tree was inferred by using the Maximum Likelihood method (ML) and General Time Reversible model (GTR). The tree is drawn to scale, with branch lengths measured in the number of substitutions per site. Evolutionary analyses were conducted in MEGA X. Scale bar indicates 0.01 (nucleotide substitution per site) sequence distance

The collected mosquitoes were grouped into 207 pools based on their species, date, and place of collection. Each of the mosquito pools was tested by PCR for the presence of flaviviruses and the only flavivirus that was detected was the ISF CxFV from the host *Cx. p. pallens*. A total of 21 pools were found positive for CxFV. The details are shown in Table 3. These mosquitoes were collected mainly from fields, nests of migrating birds, and ground water, mostly in the Jungang-dong area and several other locations in the Hado-ri and Seohong-ro areas of Jeju. The CxFVs from *Cx. p. pallens* were identified using species-specific RT-PCR targeting NS5 gene. The remaining pools with 12 other mosquito species were negative for flaviviruses using RT-PCR. The nucleotide sequences detected in all the RT-PCR positive samples were found to be 96–98% similar with sequences retrieved from GenBank (181–212 nucleotides). An NCBI BLAST analysis for each sequence showed that the top-ranking hit was that of CxFVs from *Cx. p. pallens* hosts (Table 4). One sample that tested positive for CxFV showed 95% similarity to *Cx. p. pallens* strain DG5, which was first reported in China in 2011 (GenBank accession no. JQ409191). A phylogenetic tree was also constructed based on NS5 (212 bp) gene sequences, which indicated that most of the CxFV isolates were clustering together forming a sister lineage which has common ancestral origin (Fig. 3). One of the isolate 4Mos P5 (MZ444122) was identified as a distant single divergent sequence (**Fig. ****3**).

**Table 3:** Detection of Culex flavivirus by RT-PCR in mosquito pools

Sl. No	Species	No. of mosquitoes collected (%)	No. of pools tested	No. of pools positive for *Culex* flavivirus	MIR*
1	*Culex pipiens pallens*	1450 (77.2)	77	21	14.5
2	*Culex bitaeniorhynchus*	5 (0.3)	5	0	0
3	*Culex tritaeniorhynchus*	30 (1.6)	9	0	0
4	*Culex vagans*	2 (0.1)	2	0	0
5	*Anopheles sinensis*	92 (4.9)	19	0	0
6	*Anopheles sineroides*	1 (0.05)	1	0	0
7	*Aedes vexans nipponii*	12 (0.6)	7	0	0
8	*Aedes albopictus*	164 (8.7)	42	0	0
9	*Ochlerotatus togoi*	54 (2.87)	17	0	0
10	*Ochlerotatus koreicus*	1(0.05)	1	0	0
11	*Ochlerotatus dorsalis*	3 (0.15)	2	0	0
12	*Mansonia uniformis*	6 (0.3)	3	0	0
13	*Armigeres subalbatus*	57 (3.0)	22	0	0

^*^Minimum infection rate (MIR) for flavivirus was expressed as number infected/1,000 tested

**Table 4:** Analysis of the sequences for the detection of *Culex* flavivirus

Mosquito pool name (GenBank Accession No.)	Collection date	Collection site	Grid coordinates	Mosquito No. in pool	Mosquito species	Reference Sequence GenBank Accession No	Sequence similarity (%)
4Mos P2 (MZ444119)	4/13/2018	Jungang-dong	33.2507° N, 126.5651° E	10	*Culex pipiens pallens*	MG602497.1	180/185 (97)
4Mos P3 (MZ444120)	4/13/2018	Jungang-dong	33.2507° N, 126.5651° E	10	*Culex pipiens pallens*	MG602497.1	184/190 (97)
4Mos P4 (MZ444121)	4/13/2018	Jungang-dong	33.2507° N, 126.5651° E	10	*Culex pipiens pallens*	MG602497.1	175/183 (96)
4Mos P5 (MZ444122)	4/13/2018	Jungang-dong	33.2507° N, 126.5651° E	10	*Culex pipiens pallens*	JQ409191.1	180/189 (95)
4Mos P6 (MZ444123)	4/13/2018	Jungang-dong	33.2507° N, 126.5651° E	3	*Culex pipiens pallens*	MG602497.1	179/185 (97)
4Mos P11 (MZ444124)	4/17/2018	Hado-ri	33.5150° N 126.8818° E	1	*Culex pipiens pallens*	MG602493.1	174/179 (97)
5Mos P13 (MZ444125)	5/17/2018	Jungang-dong	33.2507° N, 126.5651° E	34	*Culex pipiens pallens*	MK422518.1	185/190 (97)
6Mos P19 (MZ444126)	6/12/2018	Hado-ri	33.5150° N126.8818° E	3	*Culex pipiens pallens*	MG602493.1	189/195 (97)
6Mos P21 (MZ444127)	6/12/2018	Seohong-ro	33.2671° N, 126.5486° E	1	*Culex pipiens pallens*	MG602497.1	189/195 (97)
8Mos P21 (MZ444128)	8/6/2018	Jungang-dong	33.2507° N, 126.5651° E	79	*Culex pipiens pallens*	MK422518.1	181/185 (98)
8Mos P22 (MZ444129)	8/6/2018	Hado-ri	33.5150° N126.8818° E	1	*Culex pipiens pallens*	MK422518.1	181/185 (98)
10PP2 Mos P8 (MZ444130)	10/4/2018	Jungang-dong	33.2507° N, 126.5651° E	47	*Culex pipiens pallens*	MK422518.1	184/189 (97)
10PP4 Mos P9 (MZ444131)	10/18/2018	Jungang-dong	33.2507° N, 126.5651° E	50	*Culex pipiens pallens*	MK422518.1	185/190 (97)
10PP4 Mos P10 (MZ444132)	10/18/2018	Jungang-dong	33.2507° N, 126.5651° E	50	*Culex pipiens pallens*	MK422518.1	184/189 (97)
10PP5 Mos P11 (MZ444133)	10/18/2018	Jungang-dong	33.2507° N, 126.5651° E	50	*Culex pipiens pallens*	MK422518.1	190/195 (97)
10PP5 Mos P12 (MZ444134)	10/18/2018	Jungang-dong	33.2507° N, 126.5651° E	29	*Culex pipiens pallens*	MK422518.1	184/189 (97)
11Mos P7 (MZ444135)	11/1/2018	Jungang-dong	33.2507° N, 126.5651° E	50	*Culex pipiens pallens*	MG602497.1	189/195 (97)
11Mos P8 (MZ444136)	11/1/2018	Jungang-dong	33.2507° N, 126.5651° E	20	*Culex pipiens pallens*	MG602495.1	189/195 (97)
11 2nd MosP2 (MZ444137)	11/15/2018	Seohong-ro	33.2671° N, 126.5486° E	6	*Culex pipiens pallens*	MG602497.1	189/195 (97)
11 2nd MosP7 (MZ444138)	11/15/2018	Jungang-dong	33.2507° N, 126.5651° E	50	*Culex pipiens pallens*	MK422518.1	184/189 (97)
11 2nd MosP7 (MZ444139)	11/15/2018	Jungang-dong	33.2507° N, 126.5651° E	50	*Culex pipiens pallens*	MK422518.1	182/189 (96)

## Discussion

Recently, several flaviviruses have been sequenced, characterized, and identified in arthropods. The slow transition of the Korean climate from temperate to subtropical may also render the Korean Peninsula an ideal biological environment for the proliferation of mosquitoes as disease vectors in the near future [19]. In this study we aimed to investigate the presence of flaviviruses in the mosquitoes, and detected the presence of ISF CxFV in *Culex* species in the urban areas of Jeju province of the ROK.

CxFV from *Cx. pipiens* was the first ISF reported in Japan (2003–2004) [3] and China (2012) [20]. Since then, several other strains of CxFV have been documented [21, 22]. The *Cx. pipiens* vector plays the crucial rule in transmitting number of viruses which can cause not only bancroftian filariasis and Japanese encephalitis [23], but also other human and animal diseases, such as Rift Valley fever, Zika, St Louis encephalitis, and dog heartworm [24, 25].

Presently, vivax malaria and DENV infections are the most common mosquito-borne infectious diseases present in ROK, and almost 1171 clinical cases of dengue fever have been reported from 2015–2019 [19]. Additionally, 129 cases of JEV infection and 33 cases of Zika virus infection were also reported during 2015–2019 [20]. Furthermore, the *Cx. pipiens* species, a vector of WNV, was also found throughout ROK [26, 27]. Thus, monitoring these disease vectors is necessary to identify the possible interaction with other flaviviruses significant to public health [26, 28, 29].

*Cx. pipiens* is also the most common mosquito species in ROK [30]. The high feeding rates of mosquitoes on mammalian hosts, including humans [31], create an urgent need to study the role of *Cx. pipiens* in flavivirus transmission. Furthermore, continuous globalization and drastic climate change are also crucial factors for the spread of mosquito-borne diseases in the Jeju region, and therefore render these mosquitoes a threat for local inhabitants [32].

In this study, we collected the mosquito samples and the most predominant mosquito species identified was *Cx. p. pallens*, which accounted for 77.2% of the total population of 1,877 mosquitoes, followed by *Aedes albopictus* (8.7%), Chinese *Anopheles sinensis* (4.9%), and finally *Culex tritaeniorhynchus* (1.6%), a vector of JEV. Previously, Lee and Hwang [33] also reported similar prevalence for *Cx. pipiens* and *Aedes albopictus*; however, the prevalence of *A. sinensis* in this study was different from their results. *Culex tritaeniorhynchus* prevalence was also lower than the values reported by them (0.4%). The differences in topography, climate, and environment of the chosen areas in the two studies may explain the discrepancy in the reported results [32].

Total 207 pools were tested among which only 21 pools showed positive result for CxFV using RT-nPCR. The percentage of positive samples is low, only 10.15% (21/207 pools). The limited number of positive samples is closely related to those reported by Bryan et al. (2005) in Vietnam (26/1122 pools, 2.3%) and Ochieng et al. (2007–2012) in Kenya where only 0.3% pools were positive for CxFV [34, 35]. Additionally, Morais-Bronzoni et al. also reported the sensitivity of nested PCR assay for flaviviral detection of low viral load samples [36].

The positive pools were identified from the *Cx. p. pallens* host at the migratory bird’s nest, field and ground water area. No other ISF and pathogenic flaviviruses were detected in our study which is very similar to those reported by Bahk et al. [37]. The abundance of *Cx. p. pallens* was mostly observed in the Jungang-dong area of Jeju that shows the predominance of this species in the urban region [37].

The host of CxFV usually shows great diversity which could influence their phylogeny. CxFV has been detected from wide range of mosquitoes such as *Cx. pipiens, Culex quinquefasciatus*, and other mosquito species around the world [38, 39]. In this study a phylogenetic analysis was performed which showed two main clades with dissimilar branch length. The first clade was mainly associated with strains/isolates from Asia and USA. *Cx. Pipiens* species (in particular *Cx. p. pallens*) was the most common host identified from this clade. All of the sequences identified in our study showed more phylogenetical resemblance with this clade. The phylogenetic analysis revealed that all the newly isolated sequences were clustered together forming a single sister lineage, but the isolates were distinct from the Chinese isolates such as ZCB10 (MK422516), DG1064 (JQ308188). Among all the newly isolated sequences only 4Mos P5 (MZ444122) isolate showed the single divergent nature. Additionally, other CxFV host such as *Anopheles sinensis* (JQ308188) [38] and *Culex quinquefasciatus* (HQ634596, HQ634594, HQ634598, and FJ502995) were also reported in this group.

The second clade group of CxFV was mostly associated with strains/isolates from Asia, America, and Africa where *Culex quinquefasciatus* (JX897906, AB488433, AB639348, KY349933, EU879060, MH719098, and GQ165808) was reported as a main host [38, 39]. Additionally, *Culex tritaeniorhynchus* (JX897904) and *Culex erraticus* (KM081647) were also reported as a host. Nevertheless, it's crucial to note, that these genotypes are mainly based on the phylogenetic study of the viral polyprotein and envelope (E) gene, whereas our phylogenetic research was done with the NS5 gene.

The prevalence of *Cx. p. pallens* was relatively higher than that of other species. The MIR of CxFV in *Cx. p. pallens* was 14.5/1000 mosquitoes. A study by Kulasekera et al., (New York, 2000) also reported the MIR of WNV 8–14/1000 mosquitoes (*Cx. pipiens* and *Cx. restuans*) [40]. The pairwise nucleotide sequence identity results for the samples tested positive for CxFV were also clearly in accordance with the recommended flavivirus identification criteria (> 84%) based on the conserved region of the partial NS5 gene [41]. The detected sequences showed 95–98% sequence identity and clustered together within CxFV clade. These results confirm the presence of CxFV in *Cx. pipiens* species on Jeju Island. A significant limitation of this study is that only partial sequences (212 bp) of conserved NS5 region were used for phylogenetic analysis. However, this study reports significant findings that we believe can provide better understanding to perform further research in this area.

## Conclusion

Previous surveys based on the Jeju area consistently showed no domestic cases of mosquito-borne flaviviruses [33, 42]. Thus, this study reports for the first time the presence of CxFV in *Cx. p. pallens* in the Jeju province of ROK. The abundance of this species in the Jeju area also indicates the possible interaction with other flaviviruses causing human and animal diseases like WNV, Rift Valley fever. Therefore, intensified monitoring and long-term surveillance studies of both the vectors and viruses are essential to elucidate interaction effect. Future studies should be performed to detect more CxFV in Jeju and other region of ROK to understand the diversity, evolutionary relationship and their genetic characteristic with other species.

## Data Availability

Data and materials are available upon request to the corresponding author.

## Abbreviations

BG: Biogents' Sentinel 2 Mosquito Trap
BL: Black light trap
BLAST: Basic Local Alignment Search Tool
CxFV: *Culex* Flavivirus
DENV: Dengue virus
ISFs: Insect-specific flaviviruses
JEV: Japanese encephalitis virus
MIR: Minimum infection rate
NCBI: National Center for Biotechnology Information
N-J: Neighbor-joining
NS5: Nonstructural gene 5
PBS: Phosphate-buffered saline
PCR: Polymerase chain reaction
ROK: Republic of Korea
RT-NPCR: Reverse transcription-based nested PCR
WNV: West Nile virus
