# Correction to: Molecular detection and identification of *Culex* flavivirus in mosquito species from Jeju, Republic of Korea

**DOI:** 10.1186/s12985-021-01639-4

**Published:** 2021-08-20

**Authors:** Shilpa Chatterjee, Choon‑Mee Kim, Na Ra Yun, Dong‑Min Kim, Hyeon Je Song, Kyeoung A. Chung

**Affiliations:** 1 Department of Internal Medicine, College of Medicine, Chosun University, 588 Seosuk‑dong, Dong‑gu,, Gwangju, 61453 Republic of Korea; 2 Department of Premedical Science, College of Medicine, Chosun University, Gwangju, Republic of Korea; 3 Department of Clinical Laboratory Science, Gwangju Health University, Gwangju, Republic of Korea

## Correction to: Virol J (2021) 18:150 10.1186/s12985-021-01618-9

Following publication of the original article [1], the authors realised that a few minor but necessary edits were needed to the text. Furthermore, the statement about Funding was inadvertently left out. Authors would like to apologise for the inconvenience and reassure the readers that the correction does not change the results of the study.

In the fifth paragraph of the Discussion, *Cx. pipiens* has been corrected to *Cx. p. pallens*, thus the first sentence now correctly reads:

In this study, we collected the mosquito samples and the most predominant mosquito species identified was *Cx. p.pallens*, which accounted for 77.2% of the total population of 1877 mosquitoes, followed by *Aedes albopictus* (8.7%), Chinese *Anopheles sinensis* (4.9%), and finally *Culex tritaeniorhynchus* (1.6%), a vector of JEV.

In the eighth paragraph, *Cx. pipiens* complex should be called *Cx pipiens* species instead, and this has been corrected.

An important statement about Funding should be added:

This research was supported by a grant from the Korea Health Technology R&D Project through the Korea Health Industry Development Institute (KHIDI), funded by the Ministry of Health & Welfare, Republic of Korea (Grant No. HI20C0369).

Authors are grateful to the funders for the support.

The original article has been corrected.
